# Myocardial infarction with non-obstructive coronary artery caused by coronary artery spasm and an increase in serum homocysteine: a case report

**DOI:** 10.1093/ehjcr/ytaf118

**Published:** 2025-03-06

**Authors:** Ayano Ikeda, Jo Akama, Yoshitsugu Ohki, Hiroyuki Kunii, Shu-ichi Saitoh

**Affiliations:** Cardiovascular Medicine, Ohara General Hospital, 6-1 Uwamachi, Fukushima 96-8611, Japan; Cardiovascular Medicine, Ohara General Hospital, 6-1 Uwamachi, Fukushima 96-8611, Japan; Cardiovascular Medicine, Ohara General Hospital, 6-1 Uwamachi, Fukushima 96-8611, Japan; Cardiovascular Medicine, Ohara General Hospital, 6-1 Uwamachi, Fukushima 96-8611, Japan; Cardiovascular Medicine, Ohara General Hospital, 6-1 Uwamachi, Fukushima 96-8611, Japan

**Keywords:** Case report, Coronary artery spasm, MINOCA, Homocysteine

## Abstract

**Background:**

Although elevated homocysteine levels have emerged as risk factors for cardiovascular diseases, the associations between homocysteine levels and coronary artery spasm are not well established. We present a case in coronary artery spasm resulted myocardial infarction merged severe high level of serum homocysteine without conventional coronary risk factors.

**Case summary:**

A 57-year-old male was referred to our hospital because of chest pain. Macrocytic anaemia with a decrease in the serum vitamin B12 concentration and an increase in the serum homocysteine concentration was appeared. Endoscopy was performed first due to concerns about gastritis or bleeding risk, followed by coronary angiography. He was diagnosed autoimmune gastritis. Coronary angiography revealed no arterial stenosis, and coronary artery spasm with ischaemic ST-T changes on the electrocardiogram appeared with the intracoronary administration of acetylcholine. Inferior myocardial infarction appeared via magnetic resonance imaging. Calcium channel antagonists and vitamin B12 were treated to improve coronary artery spasm and homocysteine levels. After 1 year with adjusted serum homocysteine levels, the patient had no coronary artery spasm, and his chest pain disappeared even with the intracoronary administration of acetylcholine.

**Discussion:**

Elevated serum homocysteine levels have a possibility to worsen coronary artery spasm. We concluded that serum homocysteine levels might be a new basis for devising strategies to prevent coronary artery spasm and that optimizing serum homocysteine levels may alleviate the underlying exacerbation of myocardial ischaemia.

**Learning points**

- This rare case suggests that homocysteine may affect the disease state of coronary artery spasm.
- To elucidate the cause of myocardial ischaemia, including coronary artery spasm, it is necessary to consider various factors other than coronary risk factors, such as gastritis or anaemia.
- Repetitive provocation tests of coronary artery spasm are important for confirming therapeutic efficacy from the perspective of changes in and cancellations of vasoactive agents.

## Introduction

Elevated levels of serum homocysteine have been associated with cardiovascular and cerebrovascular diseases and increased atherosclerosis by causing endothelial layer injury, promoting inflammation, and increasing oxidative stress.^1,2^ The presence of VB6 and VB12 is required for the degradation of homocysteine, and the serum homocysteine levels increase in the absence of these vitamins. In general, high levels of serum homocysteine are present when the concentration exceeds 15 µM.^3^ Whether an increase in plasma homocysteine alone induces coronary artery spasm is unknown. We present a rare case of myocardial infarction with non-obstructive coronary artery (MINOCA) syndrome induced by coronary artery spasm with a severe increase in the serum homocysteine level (over 100 µM) under vitamin B12 deficiency anaemia.

## Summary figure

**Figure:**
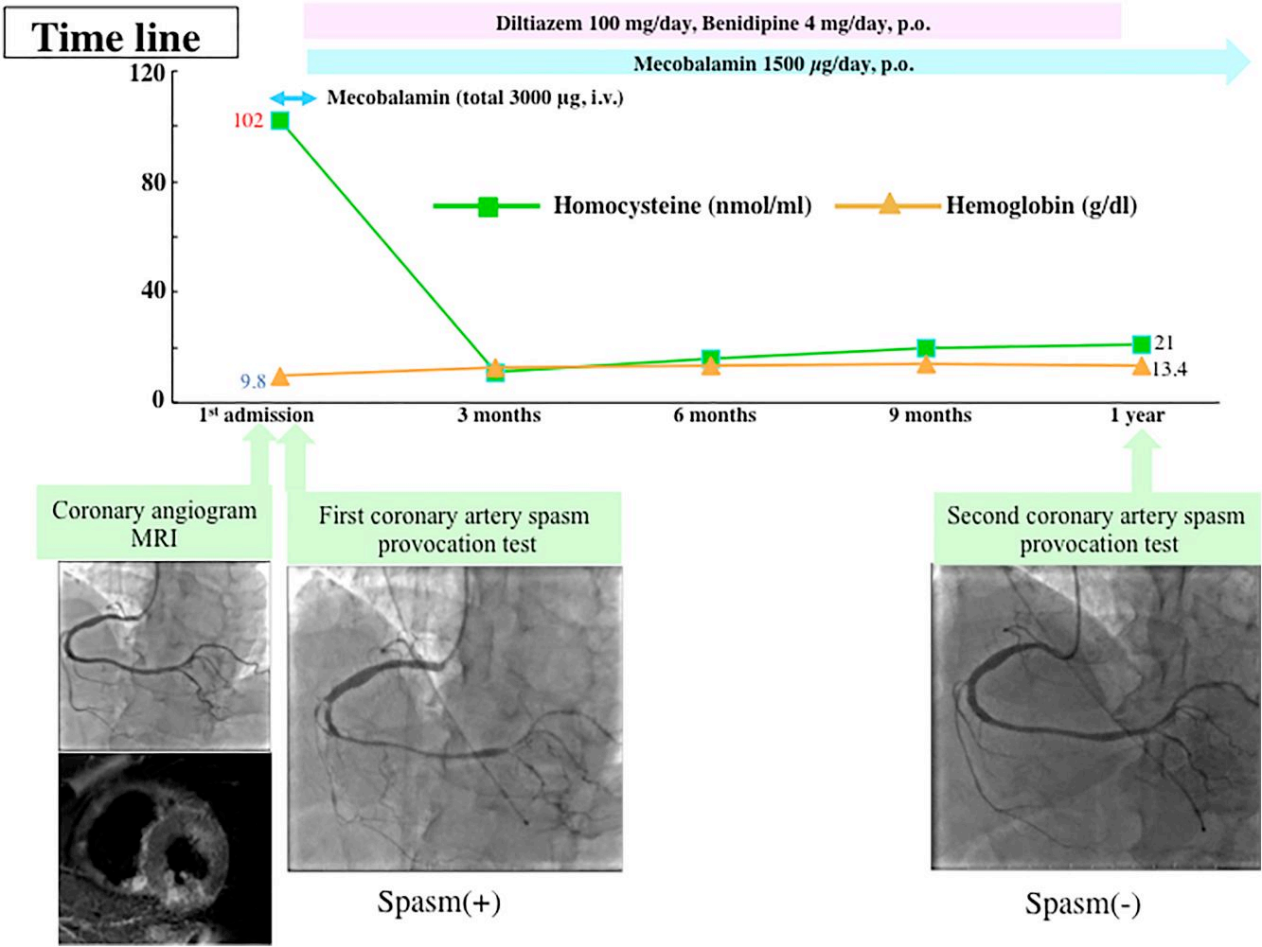


## Case

**Figure 1:**
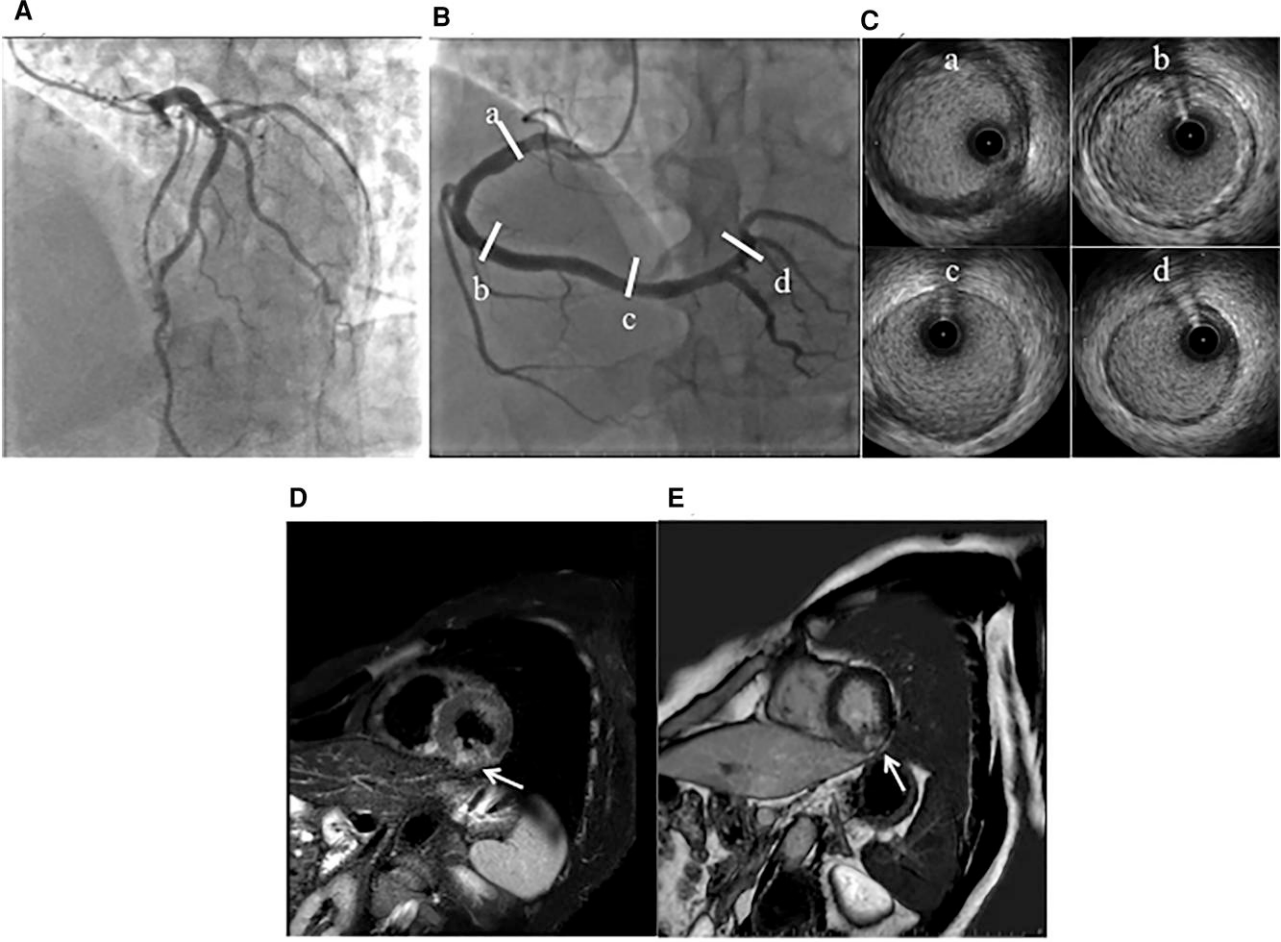
Coronary angiography and cardiovascular magnetic resonance imaging during the first hospitalization. There was no organic stenosis in the coronary artery after the intracoronary administration of isosorbide dinitrate (*A* and *B*). We subsequently performed intravascular ultrasonography of the right coronary artery, and evidence of thrombosis, plaque rupture, and coronary artery dissection was not observed (*C*). (*A*) Angiography of the left coronary artery from the LAO-cranial view. (*B*) Angiography of the right coronary artery from the LAO-cranial view. (*C*) Intravascular ultrasonography of the right coronary artery. The marks a, b, c, and d represent the intravascular ultrasonography position of the right coronary artery, as shown (*B*). In the short-axis cardiovascular magnetic resonance images, increased signal intensities in the T2-weighted short *τ* inversion recovery black-blood image (arrow) indicate recent myocardial oedema or inflammation (*D*). Late gadolinium enhancement imaging revealed irreversibly damaged myocardium in the same area (*E*, arrow). A diagnosis of acute or sub-acute inferior myocardial infarction was established.

**Figure 2:**
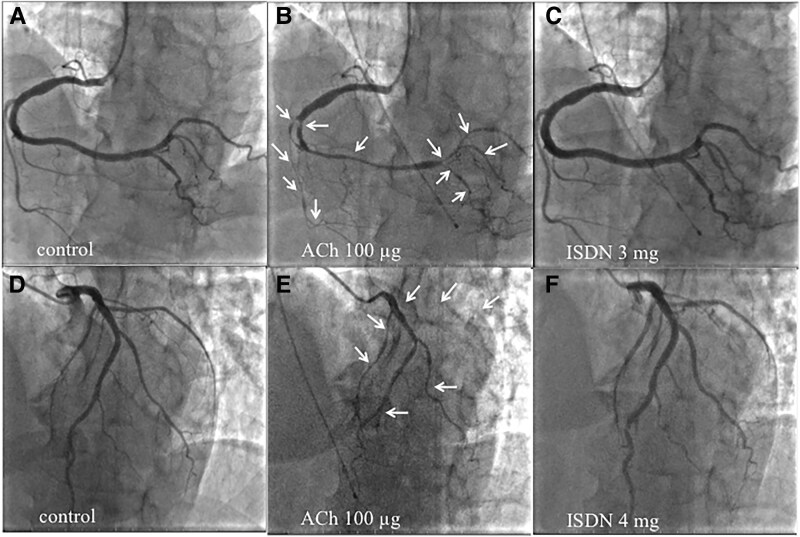
Provocation testing for coronary artery spasm with intracoronary acetylcholine during the first hospitalization. There was no obvious stenosis in the right coronary artery (*A*) or left coronary artery (*D*) on coronary angiography. Intracoronary administration of acetylcholine induced nearly complete occlusion of the right posterior descending artery, right posterolateral branch, and acute marginal branch in the right coronary artery (*B*) (arrow), and total occlusion of the left circumflex artery and diffuse narrowing of the left anterior descending coronary artery were revealed (*E*) (arrow). The chest pain and ischaemic ST-T changes in the electrocardiogram were reproduced simultaneously. These coronary artery spasms, chest pain, and ischaemic events on the electrocardiogram resolved after an intracoronary administration of isosorbide dinitrate (*C* and *F*).

**Figure 3:**
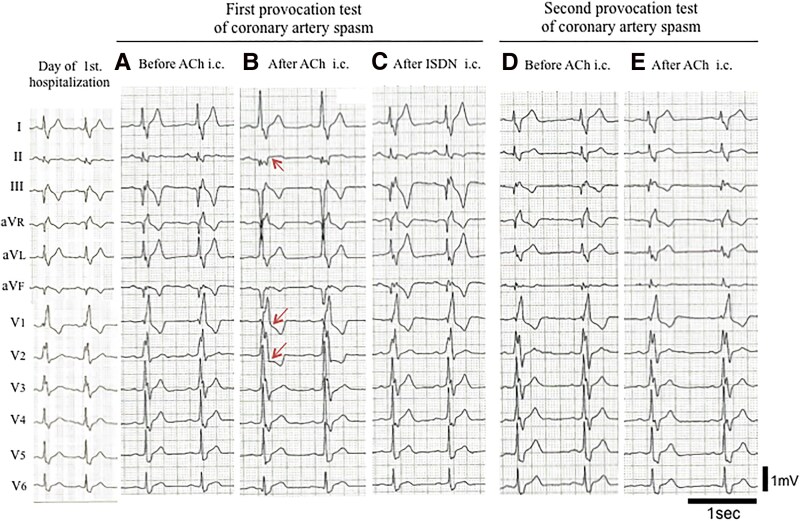
Changes in the ST-T segment of the electrocardiogram with intracoronary administration of acetylcholine. On the first provocation test for coronary artery spasm, a 1 mm ST-T segment elevation in Lead II and a −1 mm ST-T depressions in V_2_ and V_3_ mm in the electrocardiogram appeared with acetylcholine administration in the right coronary artery (*B*) (arrow) compared with the corresponding findings before administration (*A*). After isosorbide dinitrate administration, these changes in the electrocardiogram were reversed to baseline values (*C*). In the second provocation test, there were no ischaemic ST-T segment changes on the electrocardiogram before (*D*) or after (*E*) the administration of acetylcholine. i.c.; intracoronary administration.

**Figure 4:**
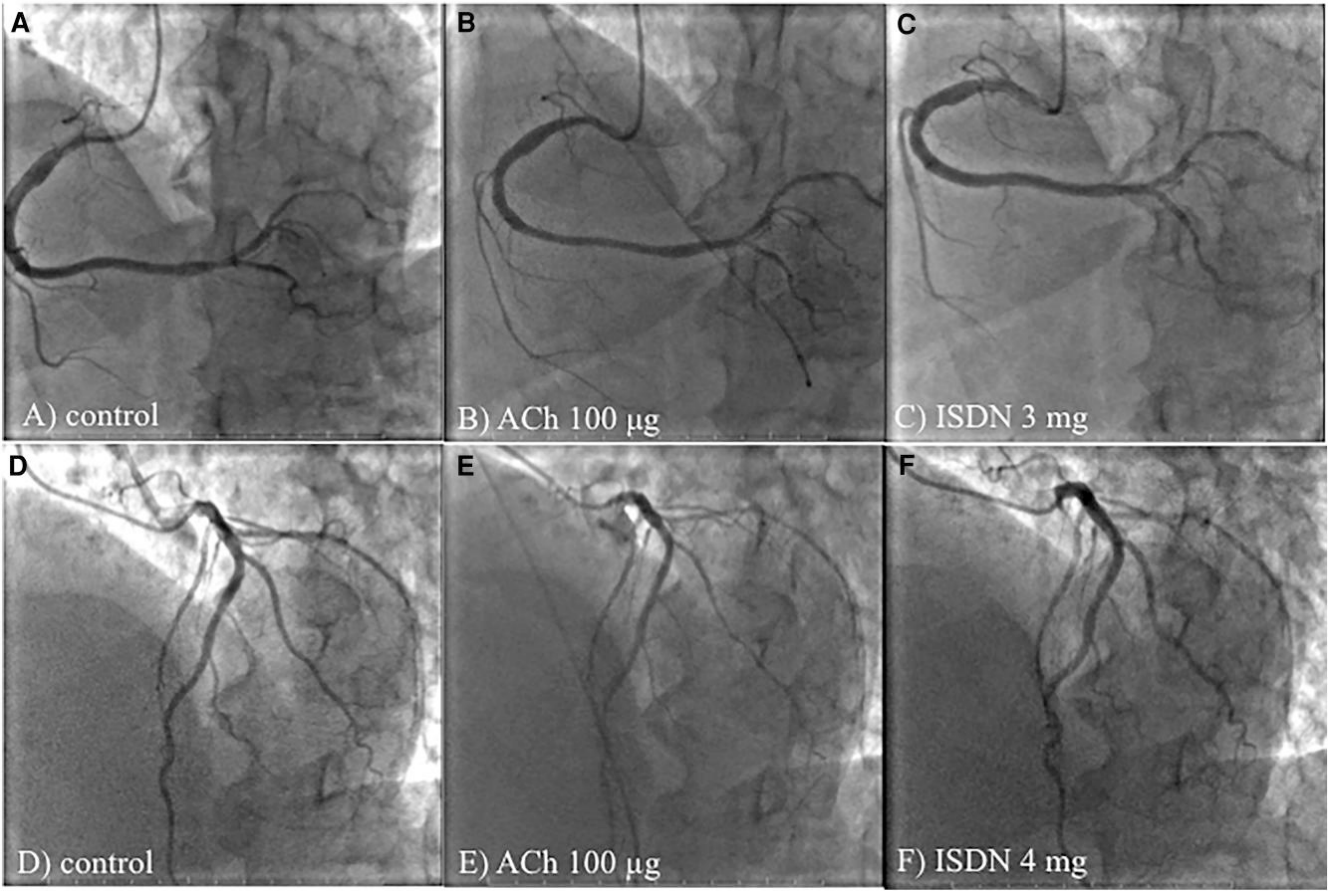
Provocation of coronary artery spasm, with the same protocol as that used in the first hospitalization and conducted after normalization of the serum homocysteine level. Coronary artery spasm did not appear after the intracoronary administration of acetylcholine (*B* and *E*). The imaging conditions in (*A, B, C, D, E,* and *F*) are similar to those in *Figure 2*.

A 57-year-old male was referred to our hospital from the clinic with suspicion of non-ST elevation myocardial infarction. The chest oppression during drinking occurred before 2 days of clinic visits, which lasted several hours and spontaneously disappeared. The patient had no symptoms at the first visit to our hospital. The patient’s pulse rate was 80 b.p.m. and regular, his blood pressure was 129/86 mmHg, and his oxygen saturation was 98% on room air. The patient had conjunctival pallor and neither venous distension nor oedema, and his cardiac and pulmonary auscultation findings were normal. The patient never smoked and had neither coronary risk factors nor a past medical history. A family history of coronary artery disease did not appear. The initial electrocardiogram (ECG) revealed a complete right bundle branch block with abnormal Q waves and negative T waves in Leads III and aV_F_. The values of serum troponin I, aspartate aminotransferase, lactate dehydrogenase, and creatine kinase (CK) were high; however, the values of CK-MB and white blood cells were within the normal ranges. In the blood test on admission, abnormal values suggesting the cause of myocardial infarction, except a low level of vitamin B12, did not appear. Therefore, we speculated that homocysteine was involved and confirmed high levels of serum homocysteine (*Table 1*). The left ventricular ejection fraction was normal (60%) according to the echocardiogram. Neither an increase in cardiac biomarkers nor worsening symptoms were observed after hospitalization. Blood tests revealed macrocytic anaemia, but haemorrhagic anaemia could not be completely ruled out without detailed inspection. We feared the worsening of anaemia and cardiac stress caused by treatment with antithrombotic agents in the case of gastrointestinal bleeding as a complication. Therefore, we prioritized upper gastrointestinal endoscopy along with medication for cardiac ischaemia in patients. The patient was subsequently diagnosed with autoimmune gastritis on the basis of a pathological examination of gastric tissue and antibody tests (positive for anti-intrinsic factor antibodies and anti-parietal cell antibodies). Coronary angiography, including intravascular ultrasonography (IVUS) at 7 days after admission, revealed no organic coronary artery stenosis, thrombosis, plaque rupture, or coronary artery dissection (*Figure 1A* and *B*). Cardiovascular magnetic resonance (CMR) imaging performed at 10 days after admission revealed irreversible damage to the myocardium, suggesting acute or sub-acute inferior transmural myocardial infarction, as shown by increased signal intensities in T2-weightened short τ inversion recovery black-blood imaging and late gadolinium enhancement imaging (*Figure 1D* and *E*). In addition, the patient did not have thrombotic disorders from not only her past history but also the results of ECG monitoring during admission, brain magnetic resonance angiography, echocardiography, and whole-leg ultrasonography. These results suggested that MINOCAs had occurred in this patient. Therefore, we again performed coronary angiography with the administration of 20, 50, and 100 μg of acetylcholine to each coronary artery to test whether coronary artery spasm was the cause of MINOCA at 12 days after admission; this assessment was in accordance with the *JCS/CVIT/JCC 2023 Guideline*.^4^ Intracoronary administration of acetylcholine induced severe constriction in the right coronary artery (RCA) and complete occlusion in the left circumflex artery (LCx), resulting in chest pain (*Figure 2*) and ischaemic ECG changes (*Figure 3*). Consequently, the administration of calcium channel antagonists (oral intake of diltiazem hydrochloride and benidipine hydrochloride) was initiated to prevent coronary artery spasm, and vitamin B12 treatment was started to normalize the serum homocysteine level. After normalization of the patient’s serum homocysteine level (1 year after his first admission), we performed coronary angiography with a coronary artery spasm provocation test using the same dose of acetylcholine. The administration of all drugs, including calcium antagonists, was stopped 3 days before the test to eliminate drug effects in accordance with the guidelines.^4^ Given that the patient tested negative for coronary artery spasm (*Figures 3* and  *4*), we discontinued the administration of all the calcium channel antagonists and continued supplying vitamin B12. The serum LDL cholesterol level was within the normal range at follow-up (80 mg/dL) without treatment with medication for dyslipidaemia. He carries nitroglycerine spray in treated clothes for temporary use. Ischaemic heart events have not yet recurred.

**Table 1:** Blood tests during admission

Tests	Day of hospitalization	Fourth day of hospitalization	Normal range
WBC (×10^3^/µL)	5.7	5.1	3.3–8.6
HGB (g/dL)	9.8	9.9	13.7–16.8
PLTS (×10^3^/µL)	250	267	158–348
MCV (fl)	121.3	120.3	83.6–98.2
MCHC (g/dL)	36.6	37.1	31.7–35.3
AST (U/L)	37	26	13–30
LDH (U/L)	462	449	124–222
CK (U/L)	300	215	59–248
CK-MB (U/L)	8.0	6.0	<10.0
TG (mg/dL)	133		40–234
HDL-C (mg/dL)	41		38–90
LDL-C (mg/dL)	79		65–163
FBS (mg/dL)	108	106	73–109
HbA1c (%)	5.4		4.9–6.0
Uric acid (mg/dL)	3.7		3.7–7.8
BNP (pg/mL)	31.2	28.9	<18.4
TnI (pg/mL)	4169.9	1646.9	<34.2
Vitamin B_1_ (µg/dL)	3.5		2.6–5.8
Vitamin B_12_ (pg/mL)	150		233–914
Folic acid (ng/mL)	5.3		3.6–12.9
Homocysteine (nmol/mL)	102		5–15

## Discussion

Homocysteine is a sulfur-containing amino acid formed during the metabolism of methionine, an essential amino acid derived from dietary proteins.^5^ Homocysteine can induce vascular damage by promoting platelet activation and vascular smooth muscle cell proliferation.^1^ In some cases, evidence of thrombosis, plaque rupture, or coronary artery dissection was not observed via IVUS examination. On the other hand, ischaemic changes are caused by the intracoronary administration of acetylcholine, which is a useful method to estimate coronary artery spasm and endothelium-dependent relaxation via nitric oxide.^5^ The pathogenesis of this event is unclear; however, we speculate on the contribution of homocysteine. The homocysteine is readily oxidized, leading to the formation of superoxide and hydrogen peroxide. Oxidative stress induces thiol oxidation in endothelial nitric oxide synthase (eNOS), which results in the attenuation of bioactive nitric oxide generation through Akt/eNOS signalling.^6–8^ The attenuation of endothelial nitric oxide activity plays an important role in the pathogenesis of coronary vasospasm.^9^ In general, severely high levels of serum homocysteine (>100 μM), such as this case, have been reported to induce vascular damage.^10^ Therefore, the impairment of nitric oxide activity by homocysteine might be a background of the cardiac event in this case. However, this finding should be supported by more literature and studies in the future.

Homocysteine is remethylated to methionine via a vitamin B12-dependent reaction. Deficiency in vitamin B12, which is involved in homocysteine metabolism, results in increased serum concentrations of homocysteine,^11^ although it is controversial whether a decrease in vitamin B12 is a coronary risk factor.^12^ There have been no reports that a decrease in vitamin B12 alone can induce coronary artery spasm. In this case, the decrease in stored vitamin B12 might have occurred due to the malabsorption of vitamin B12 by autoimmune gastritis and may have led to increased homocysteine due to remethylation disorders.^13^ Previously, patients with coronary artery spasm with abnormal values of serum homocysteine and vitamin B12 were reported.^14^ However, whether elevated serum homocysteine or vitamin B12 levels trigger coronary artery spasm is controversial because the increase in homocysteine levels is small (14.8 ± 5.3 µM), and those patients have multiple coronary risk factors, such as diabetes, hypertension, dyslipidaemia, and smoking. On the other hand, the patient in our case did not have the traditional risk factors for vasospasm^4^: smoking, high blood pressure, obesity, impaired glucose tolerance, dyslipidaemia, and heavy drinking (*Table 1*). In any case, further study is needed to determine the contribution of vitamin B12 to coronary artery spasm.

The level of haemoglobin in the peripheral blood at the time of admission was 9.8 g/dL, and there was no history that could be inferred as the cause of anaemia. Blood tests revealed macrocytic anaemia, but haemorrhagic anaemia could not be completely ruled out without detailed inspection. If coronary artery intervention with stenting is performed, treatment with two types of antiplatelet agents is required to prevent stent thrombosis. The worsening of anaemia caused by treatment with antithrombotic agents in the case of gastrointestinal bleeding as a complication may induce fatal complication death.^15^ The reason 7 days were needed for the detailed examination with coronary angiography was the problem of co-ordination with other departments (e.g. gastroenterology). We speculate that the onset of myocardial infarction occurred 2 days before admission. There was no obstruction in the RCA with coronary angiograms, although the CMR images suggested transmural myocardial infarction. It cannot be ruled out that reperfusion in the RCA occurred before the coronary angiogram. We will perform coronary angiograms as soon as possible in such cases, with safety in mind from the next time.

Supplementation with vitamin B12 normalized the serum homocysteine level, probably due to the optimized metabolism of methionine, and the second coronary artery spasm provocation test was negative after 1 year.^16^ Thus, we believe that homocysteine normalization results in clinical improvement, although further study is needed.

## Conclusion

We concluded that an optimized serum homocysteine level might play an important role in preventing myocardial ischaemia, including coronary artery spasm.

## Data Availability

The corresponding author, upon reasonable request, will share all the data relevant to this case.

## Lead author biography

Ayano Ikeda, MD, is a specialist for internal medicine and is working at the Department of Cardiology, Ohara General Hospital, Fukushima, Japan.

## Notes

**Consent:** The authors confirm that written consent for the submission and publication of this case report, including images, values of examination, and associated text, has been obtained from the patient in accordance with COPE guidance.

**Funding:** None declared.
